# Examination Questions, Pennsylvania State Board of Veterinary Medical Examiners

**Published:** 1896-07

**Authors:** 


### SOCIETY PROCEEDINGS.

### PENNSYLVANIA STATE BOARD OF VETERINARY MEDICAL EXAMINERS.

### EXAMINATION QUESTIONS, PENNSYLVANIA STATE BOARD OF VETERINARY MEDICAL EXAMINERS, AT PHILADELPHIA, JUNE 15 AND 16, 1896.

APPLICANT to answer ten of the questions on each branch, but no more. Check the number of each one of the questions you have answered. Unless otherwise stated, all questions to relate to the horse.

*Practice of Veterinary Medicine.* 1. Give the causes, symptoms, and treatment of polyuria. 2. Give the diagnostic symptoms of pleurisy and treatment of the same. 3. Give the symptoms of and exciting causes of tetanus. 4. Give the symptoms of choke in the horse and ox, and treatment for the same. 5. Give the diagnostic symptoms of impaction of the stomach, and treatment for the same. 6. Give the symptoms, causes, and treatment of osteoporosis. 7. How would you distinguish between contagious pleuro-pneumonia and tuberculosis in the ox? 8. Give the diagnostic symptoms of hog cholera, and the best methods of control of such an outbreak. 9. What group of general symptoms make it difficult to distinguish between intestinal parasites and distemper in the dog in the incipient stage? 10. What is periodic ophthalmia? How would you diagnose it? and state treatment. 11. What are the predisposing causes, diagnostic symptoms, and treatment of parturient apoplexy (milk fever) in cattle? 12. What is influenza, and what forms and group of symptoms does it assume? 13. In an outbreak of rhino-adenitis among a large number of animals, what general plans of management would you adopt? 14. What varieties of worms infest the digestive tract of a dog? 15. What diseases infect the air-passages of sheep?

*Sanitary Medicine, Meat- and Milk-Inspection.* 1. What diseases of animals are most dangerous to consumers of meat? 2. What measures are required to suppress and eradicate pleura-pneumonia contagiosa and tuberculosis in cattle? 3. Give the symptoms and sanitary requirements of glanders. 4. In what manner would you demonstrate the contagiousness of rabies? 5. What sanitary conditions should be observed in the construction and management of a large barn where transient animals are sheltered? 6. Under what conditions would you condemn meat for human food? 7. How would you impress your seal of condemnation of live stock at export stations? 8. Supposing that you were placed in charge of some large abattoir as inspector. Explain how you would conduct the affairs of your office? 9. What are the indications of putrefaction? 10. How would you examine pork for trichinæ? 11. What is colostrum? Give its composition in the cow. 12. What would you consider a proper basis of solids in milk? How would you determine same? 13. Under what conditions would you condemn milk as unfit for human food? 14. What is the specific gravity of cow's milk of good quality? 15. What three essential parts is milk composed of, and how may these principal constituents be separated?

*Zoötechnics.* 1. Describe the character of an eight-year-old mouth. 2. What is atavism? Give an example. 3. Give the signs of a good milk cow. 4. What percautions would you observe in purchasing a horse for breeding purposes? What diseases are hereditary, and what is the relative value of pedigree and performance? 5. What is a normal food, and what constitutes a normal day's ration for a horse? 6. Describe a Dutch (Friesian) cow. 7. What are the races of horse (equinus caballus). Name and describe two. 8. Define and illustrate by example the different methods of reproduction applied to the domestic animals. 9. Give the difference between the Spanish and the oriental ass. 10. What, in your opinion, is the relative influence of the male and female? 11. What is hybridization? Give hybrid between equine and asinine species. 12. What are the essential characteristics of a hybrid? 13. Define precocity, its causes and characteristics, with example. 14. Describe the time and frequency of the menstruations in the mare and cow. 15. What age does puberty occur in the various domestic female animals?

*Therapeutics and Materia Medica.* 1. What are the therapeutic uses for camphor? 2. What is the dose for a horse of oleum tiglii? Hydrargyrum chloridum mite? 3. Describe the physiological action of opium. What is the antidote for opium-poisoning? 4. Name the alkaloids of hyoscyamus. 5. What are the uses of chloral hydrate and the contra-indications to its use? 6. Write a prescription for purpura. 7. What are the therapeutic uses of belladonna? Give the dose of its principal alkaloid to a setter dog. 8. What is the antidote for lead-poisoning? 9. Describe briefly the physiological action and uses of lobelia. 10. What do you consider the best cardiac depressant? Anti-spasmodic? Alterative? 11. What are the contra-indications to the use of chloroform? 12. Describe briefly how you would treat a case of arsenical poisoning in a dog. 13. How would you disinfect a stable? 14. Name three mineral astringents. Three vegetable astringents. 15. What is the principal use of spigelia?

*Veterinary Diagnosis.* 1. How would you differentiate between chronic glanders with lesions in the head and collection of the nasal sinuses? 2. What are the characteristic symptoms of spavin? 3. Give the diagnostic signs of pleurisy and pneumonia in their several stages. 4. What are the diagnostic signs of traumatic pericarditis in cattle; for what other pathological conditions may it be mistaken? 5. How would you distinguish mange from vesicular eczema in the dog? 6. What are the diagnostic symptoms of collection of the guttural pouches and with what other conditions may it be confounded? 7. How do you differentiate between shoulder and foot lameness? 8. Upon what is the diagnosis of navicular arthritis based? 9. In case of lameness, how do you determine the lame member? What is the difference in the movements of the member in shoulder lameness as compared with foot lameness? 10. What are the diagnostic signs of laminitis? 11. Give a chronic case with a history of gradual falling off in flesh, tucked-up abdomen, general stiffness in locomotion, or lameness, possibly intermittent. Of one or more members, what would be your diagnosis? With what other conditions may it be confounded? 12. What is your general procedure in examining a horse for lameness under the most favorable conditions? 13. How would you diagnose anthrax in cattle? 14. How do you administer tuberculin for diagnostic purposes in tuberculosis, and what constitutes a reaction? 15. What constitutes a typical reaction with mallein in a glandered horse?

*Chemistry.* 1. Define chemistry. 2. Define an element. 3. What is atomic weight? 4. Name and define the various kinds of attraction. 5. What are the symbols for the following elements: Oxygen, mercury, silver, lead, sodium, manganese, potassium, magnesium, nitrogen, and iron? 6. What is a test for a carbonate? 7. What is the composition of water? Air? 8. Write the formula for common salt, hydrochloric acid, sulphuric acid, Epsom salt, and baking soda. 9. What is the atomic weight of hydrogen, oxygen, nitrogen, sulphur, and silver? 10. What is the flame-test for sodium? Copper? 11. Give a test for arsenic. 12. Give a test for albumin in the urine. 13. Give a test for sugar in the urine. 14. How would you prepare sulphuretted hydrogen? 15. How would you test a case of lead-poisoning in a cow from its milk?

*Pathology.* 1. Define inflammation. 2. What is a complete cell? 3. What are the steps to inflammation? 4. What are the terminations of inflammation? 5. What are the two general divisions of inflammation? 6. What is chronic inflammation? 7. Define glanders. What kind of an inflammation? 8. Define tuberculosis. 9. What is a cyst? How many kinds? 10. What is a malignant tumor? Benign tumor? 11. What are the physical types of the following tumors: Osteoma, myoma, neuroma, lipoma? 12. What is leukæmia? 13. What is the cause of moist gangrene? 14. What are the stages of croupous pneumonia? 15. Define anthrax.

*Obstetrics.* 1. What does successful fecundation depend upon? 2. Mention some of the causes of sterility. 3. Describe fœtal circulation. 4. What are the uses of the liquor amnii? 5. What constitutes the umbilical cord and what purpose does it serve? 6. Give some of the rational and some of the material signs of pregnancy. 7. What is the normal duration of pregnancy in the mare? What in the cow? 8. What hygienic measures would you observe in the management of animals during gestation? 9. Name the diseases peculiar to pregnancy. 10. Mention some of the accidents of pregnancy. 11. What constitutes normal parturition? 12. What are the various stages of parturition? 13. How would you deliver a mare or cow in which the four feet of the fœtus were presented sterno, abdominal presentation, head retained? 14. How would you treat complete inversion in the uterus? 15. What is mammitis? Give symptoms and treatment for same.

*Surgery.* 1. Describe symptoms and surgical treatment for collection of the superior maxillary sinuses in the horse. 2. Describe symptoms and surgical treatment of collection of the guttural pouches in the horse. 3. Describe symptoms and surgical treatment of roaring in the horse, viz.: Laryngotomy or arytenectomy. 4. Describe symptoms and surgical treatment for cartilaginous quittor, resection of lateral cartilage. 5. Describe symptoms and surgical treatment for amputating penis of the horse. 6. Describe symptoms and surgical treatment of spavin. 7. Describe symptoms and surgical treatment for paraphimosis. 8. Describe symptoms and surgical treatment for navicular disease. 9. Describe symptoms and surgical treatment for ringbone. 10. Describe symptoms and surgical treatment for conjunctivitis. 11. Describe symptoms and surgical treatment for string-halt. 12. Describe surgical treatment for toe crack, superficial and deep. 13. Give indications for tracheotomy in the horse—describe operation. 14. Describe ovariotomy in the bitch and give physical contra-indication. 15. Describe symptoms and surgical treatment for impaction of the rumen of the cow.

*Anatomy.* 1. Describe the bones of the pelvis. 2. Describe a dorsal vertebra. 3. Describe the flexor pedis perforans and its check ligament. 4. Name the superior cervical muscles in their order of superposition. 5. Name and describe the ligaments of the fetlock. 6. Name and locate the principal superficial veins of the horse. 7. Describe the plantar nerves. 8. Describe the regional anatomy in low neurectomy. 9. What is the difference between the lungs of the horse and those of the ox, and what the principal peculiarity of the latter? 10. Describe the arterial supply of the foot below the fetlock. 11. Describe the cunean tendon. How can it be located? 12. Describe the liver. 13. What is the difference between the kidney of the horse and that of the ox? 14. Describe the common carotid arteries. 15 Describe the pneumogastric nerve.

