# Correction

**Published:** 1896-08

**Authors:** 


### CORRECTION.

THE July number of the JOURNAL inadvertently credited the name of R. G. Rice, V.S., who had successfully passed the Pennsylvania State Board of Veterinary Medical Examiners, to the Ontario Veterinary College. Dr. Rice was graduated from the class of '96, New York College of Veterinary Surgeons.

